# Does Q223R Polymorphism of Leptin Receptor Influence on Anthropometric Parameters and Bone Density in Childhood Cancer Survivors?

**DOI:** 10.1155/2013/805312

**Published:** 2013-11-04

**Authors:** Malgorzata Sawicka-Żukowska, Maryna Krawczuk-Rybak, Katarzyna Muszynska-Roslan, Anna Panasiuk, Eryk Latoch, Jerzy Konstantynowicz

**Affiliations:** Department of Pediatric Oncology and Hematology, Medical University of Bialystok, 15-274 Bialystok, Poland

## Abstract

Childhood cancer survivors are in augmented risk for developing obesity. For many factors leptin and leptin receptor gene polymorphism play an important role in the development and metabolism not only of fat, but also, bone tissue. The aim of the analysis was to find the relationships between Q223R, leptin levels, and anthropometric parameters. *Patients and Methods*. In the study 74 cancer survivors participated (ALL *n* = 64, lymphomas *n* = 10), and the control group consisted of 51 healthy peers. Leptin blood concentration was determined by ELISA method. To estimate leptin receptor gene polymorphism, RFLP method was used. Bone mineral density (BMD) and content (BMC), fat, and lean tissue measurements were obtained by DXA. *Results*. We found no correlations between serum leptin concentrations and anthropometric parameters nor BMD. Serum leptin concentrations were significantly lower in the group of cancer survivors compared to controls; however, in those overweight from examined group we found leptin levels higher than those in nonoverweight. Genotype Q223R was not associated with higher leptin levels, BMI, BMD, body fat or lean tissue. *Conclusion*. To our knowledge, this is the first report describing the relationship between BMD and Q223R polymorphism in childhood cancer survivors. Further analysis, based on a larger group of patients, is needed to confirm these findings.

## 1. Introduction

Fat tissue is a source of many significant cytokines, such as leptin, adiponectin, and resistin, affecting metabolism, energetic balance, and modifying peripheral insulin sensitivity of tissues, as well as carbohydrate metabolism. Cytokine with mostly multipotential performance is leptin, encoded by the so-called obesity gene—OB, which affects body mass regulation through hypothalamus influence on appetite and energy expenditure. Leptin is a 16 kDa molecular weight substance with pleiotropic action. It is involved in many significant life processes like metabolism, hematopoiesis, and angiogenesis and in pubescence and reproductiveness [1, 2].

*In vitro* studies had demonstrated that leptin stimulates human bones stem cells for differentiation into osteoblasts, increases the mineralization of bone tissue, and inhibits osteoclast genesis. Possibly, leptin stimulates ontogenesis and inhibits resorptive processes in bone tissue. Research *in vivo* have shown that the deficiency of leptin in *ob/ob* mice or *db/db* mice leads to an increase in bone mineral density. It is commonly known that the final effect of leptin's action is the result of its peripheral—positive and central—negative performance and depends on its concentration in serum. Both peripheral and central effects can timely equilibrate. In cases of obesity with hyperleptinemia and central insensitivity for leptin, dominant is the beneficial peripheral effect, which consequently leads to higher bone mineral density among patients [3–6]. One of the important factors regulating leptin's concentration is its soluble OB-Rb receptor, functioning as leptin-binding protein. Genetic screening of leptin receptor gene polymorphism is not influenced by leptin concentration changes (for example, after meal consumption) [3, 4]. 

Few leptin receptor (LEPR) genes polymorphism were identified, among them Q223R (Gln223Arg), K109, and K656R. Gln223Arg polymorphism is characterized by adenine on guanine substitution in 668 position of the 6th exon, which is followed by transmembrane permeability and the modification of functions. Relation between carrier state of Gln223 (A) allele versus high leptin concentration in blood serum and body mass index (BMI); bone mass, bone mineral, and fat tissue content were described [2, 3, 7–9].

In children after completed anticancer treatment, there is a possibility of overweight occurrence and obesity as well as height deficiency, reduced bone density, and abnormal mineralization of bone tissue. Those disorders are caused by the disease itself, especially in the cases of leukemias and non-Hodgkin's lymphomas but also by long-standing steroid therapy, chemotherapy, central nervous system irradiation, eating disorders, low physical activity during and after the treatment, and by many other environmental factors to which patients can be exposed. Components of metabolic syndrome are observed much more often in children after antitumor treatment than in the population of healthy peers [10, 11].

Attempts of explaining correlation between leptin concentration, polymorphism of the gene of leptin's receptor, and bone tissue density were examined in the last decade by many authors. Most of the results are based on analyzing healthy adults and only few on children with disorders such as obesity, anorexia nervosa, human growth hormone deficiency, or rheumatological conditions. The aim of our research was to distinguish the negative environmental factors from the influence of the single genetic factor on anthropometric and densitometry parameters in children after completed antitumor treatment [12–17].

## 2. Patients and Methods

74 Caucasian patients (42 boys) from the Department of Pediatric Oncology and Hematology of the Medical University of Bialystok were examined after the completed antineoplastic treatment for acute leukemia (*n* = 64) and lymphomas (NHL; *n* = 10) (Table 1). Oncological treatment was performer in years 2000–2006. Only children aged 10 years old and older were qualified to the analysis because of the required cooperation during densitometry (DXA). Control group for the data on LEPR genotype and for estimation of the concentration of leptin in blood serum was recruited from 51 nonobese patients (34 boys) hospitalized in the department due to reasons other than neoplastic diseases. 

Bioethical Committee of the Medical University of Bialystok gave the permission to perform the analysis. Parents and guardians of each patient have signed the written consent to participate in the examination.

### 2.1. Anthropometric Parameters

Measurements of each anthropometric parameters at single patient were performed by one trained person. Body mass index was calculated using the following formula:

(1) $$\begin{array}{c} \text{BMI}\;\left[\text{kg}/{\text{m}}^{2}\right]=\text{body}\;\text{mass}\;\left[\text{kg}\right]/{\left(\text{height}\;\left[\text{m}\right]\right)}^{2}. \end{array}$$

Obtained results were computed into SDS values [18]. Measurements of waist and hip circumference were made by standardized tape-measure (in centimeters); and waist to hip ratio (WHR) was determined by the following formula: waist circumference (cm)/hip circumference (cm), where the value 0.85 was cut-off value. 

### 2.2. Body Composition Parameters Evaluation

Measurements of total body fat mass (FM), lean mass (LM), bone mineral content (BMC), and total bone mineral density (total BMD) as well as in spine bone mineral density (spine BMD) were performed with dual energy X-ray densitometry (DXA) using GE-Lunar equipment. To gain reliable results, because of age diversity among examined patients and huge substantial differences in measurements, the values of both densitometry and anthropometric parameters were computed in SDS. 

### 2.3. Evaluation of Leptin Concentration in Blood Serum

To valuate leptin's concentration in blood serum (ng/mL) ELISA method with Human Leptin Human Leptin Quantikine Kit (R&D Systems, USA and Canada) was used. 

### 2.4. Polymorphism Q223R of Leptin Receptor Gene

In evaluating polymorphism of leptin receptor gene, the method of restriction fragment length polymorphism (RFLP) with polymerase chain reaction (PCR) was used. Samples of stabilized peripheral blood EDTA were taken from every patient and stored in the temperature of −20°C degrees. To isolate genomic DNA MasterPure TM DNA Purification Kit was used, following manufacturer's instructions. Amplification of gained DNA (in the capacity of 0.2 microl) was performed in solution of 20 microliters of final capacity, with the consistence of 0.2 microliter of every primer (forward 5′ AAACTCAACGACACTCTCCTT 3′, reverse 5′ TGAACTGACATTAGAGGTGA 3′) and 10 microns of Taq polymerase (JumpStart TM REDTaq TM DNA Polymerase by Sigma). Allele-specific polymerase chain reaction was performed in MJ Mini Personal Thermal Cycler (BioRad) according to the following scheme: initial denaturation in 94°C for 4 minutes, thereafter 35 cycles in 94°C for 40 seconds, primer binding in 55°C for 40 seconds, and elongation in 72°C. For separation of reaction's products electrophoresis in agarose gel with ethidium bromide was used. Obtained bands were evaluated in ultraviolet lamp.

### 2.5. Statistical Analysis

Statistical analysis was performed with the STATISTICA 10.0. by StatSoft Inc. Longitudinal values were compared between examined groups with Student's *t*-test. For comparison of genotypes groups ANOVA test was used. The Hardy-Weinberg principle was used to rate the genotype distribution among the examined population. Value *P* < 0.05 was assumed as a statistically significant. 

## 3. Results

### 3.1. Distribution of Q223R Leptin Receptor Gene

Distribution of Q223R in examined and control group is shown in Table 2. As we expected, in our analysis both groups (examined and control) presented the majority of heterozygotes with GA genotype. Despite of no statistically important differences in distribution, the examined group higher percentage of GA genotype heterozygotes (56.8%) than the control group (47.05%), where the balance is moved towards often occurrence of G alleles, although the percentage distribution of AA homozygotes is highly comparable in both groups. Obtained distribution of particular genotypes in examined and control groups was comparable with the expected, based on Hardy-Weinberg principle. Genotype distribution after the division of the group into subgroups by sex and diagnosis showed the predominance of heterozygotes, similar to the whole group, with the exception of girls from control group, in which GG homozygous genotype was the dominating one (Table 2).

### 3.2. Anthropometric Parameters

Values of the anthropometric and densitometric parameters in the whole group, according to Q223R LEPR genotype were shown in Table 3. Different Q223R LEPR genotype did not correlate with neither height, body mass index, WHR, total bone mineral density, spine bone mineral density, fat mass, lean mass, nor bone mineral content expressed as SDS.

### 3.3. Overweight/Obesity

**Figure 1:**
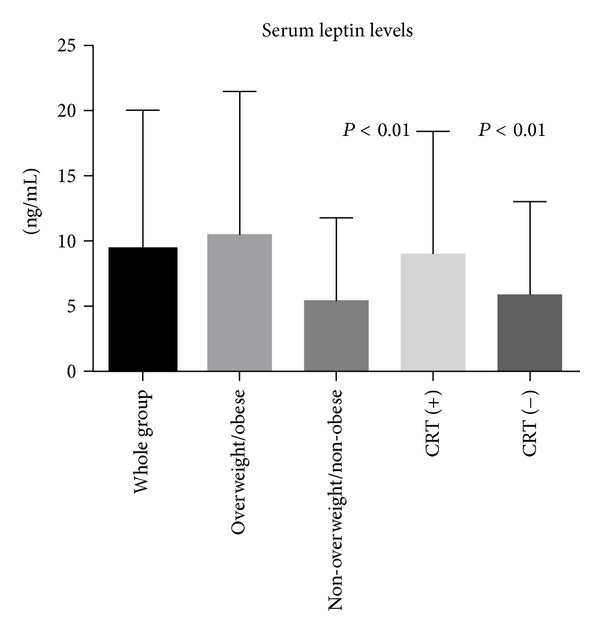
Serum leptin levels in the whole group, overweight/obese and nonoverweight/obese subjects (*P* < 0.01), and patients who receive and who did not received cranial radiotherapy (*P* < 0.01). CRT (+)—patients who received cranial radiotherapy; CRT (−)—patients who did not receive cranial radiotherapy.

Total percentage of children with overweight and obesity defined by WHO definition (BMI > 24 kg/m^2^) in analyzed group was 18.91%: overweight—*n* = 12, 10 boys (16.21%) and obesity—*n* = 2, 2 boys (2.7%). The whole group of children with BMI > 24 kg/m^2^ (*n* = 14) showed the predominance of GG allele (50%) over AG allele (42.85%) and AA (7.15%). LEPR genotype of both obese patients was GG. Twelve of 14 patients in this group were boys (*P* < 0.05). Leptin levels in overweight patients compared to nonoverweight were statistically higer (10.572 ± 10.853 ng/mL versus 5.462 ± 6175 ng/mL; *P* < 0.0005) (Figure 1). Values of WHR were within normal rate only in 4 from 14 children (33.3%) in group with elevated BMI, compared to nonoverweight children. WHR in overweight and obese group was statistically higher than in nonoverweight group (median 0.8950 (0.7600–0.9600) versus 0.8350 (0.6200–1.100); *P* < 0.0001).

### 3.4. Cranial Radiotherapy

16 individuals (5 girls) received cranial radiotherapy. Thirteen received dose of 12G and 3 dose of 18G, according to treatment protocols. The distribution of LEPR Q223R genotype in irradiated patients was AA—1 (6.25%), AG—10 (62.5%), and GG—5 (31.25%). Three from these 16 patients had BMI > 24 kg/m^2^, two presented AG genotype and one genotype GG. Serum concentrations of leptin in the group of individuals who received CRT were statistically higher than in group without radiotherapy (*P* < 0.01) (Figure 1).

### 3.5. Leptin Concentrations

Leptin concentration in all examined group was significantly lower than in control group. There were no statistically significant differences in leptin concentration between particular genotypes. We did not find any differences in leptin concentrations between girls and boys nor in children with a different Tanner stage. In the comparison between the genotypic subgroups, significantly lower concentrations of leptin in homozygous GG groups (*P* = 0.021) were noticed. AA homozygotes presented the opposite relationship (*P* = 0.031) (Table 4). 

## 4. Correlations

In our analysis we did not find any correlations between leptin concentrations and anthropometric parameters such as height, body mass, WHR, BMD, BMC, FAT, and LEAN tissue in the whole group, as well as in subgroups with three different LEPR genotypes. All of the correlations are showen in the Tables 5, 6, 7, and 8. 

## 5. Multivariate Analysis

To estimate the factors which influence the risk of obesity the model of forward stepwise regression for BMI was prepared. Strong positive connection (adjusted *r* ^2^ = 0.8858, *F* = 28.1607, *P* = 0.00002) was found between BMI and fat mass and total bone mineral density (Table 9). Other parameters such as age of the analysis, gender, Tanner stage, CRT, LEPR genotype, leptin levels, lean mass, and bone mineral content did not influence BMI in this model. 

To find the factors influencing leptin levels forward stepwise regression was also used. Analysis showed strong positive influence (adjusted *r* ^2^ = 0.7577, *F* = 9.7615, *P* = 0.0019) of bone mineral density on leptin levels, whereas negative influence of spine bone mass. Similarly, the lean mass influences leptin concentrations negatively. Carriage of the GG LEPR genotype and cranial radiotherapy were also included in the model, amplifying it (Table 10). No influence of age, gender, age of diagnosis, other anthropometric and densitometric parameters on leptin levels was shown.

## 6. Discussion

Distribution of particular genotypes in presented analysis is similar to the previous analysis performed in our department on a group of 95 children—52 diagnosed with neoplastic disease (GG—50%, AG—21.2%, AA—21.2%) and 43 healthy peers (GG—39.5%, AG—48.8%, AA—11) [19]. Distribution of particular Q223R polymorphism genotypes indicates notably ethnic differences. Ragin et al. performed the valuation of Q223R LEPR genotypes distribution in different ethnic groups containing 1400 individuals. Percentage value of the homozygous A allele carriers from the examined group resembles almost perfectly the one from the analysis performed by Ragin et al. (AA = 14.9% versus 14.16%) and is only slightly lower in control group (13.7%) [7].

 Analyzing the whole group of cancer survivors we did not find any correlations between leptin levels and anthropometric parameters like BMI, WHR, fat, and lean mass or BMD, although we found statistically higher concentration of leptin among those individuals with BMI over 24 kg/m². Similar to us, Komşu-Örnek et al. analysis found no correlations between leptin concentrations and LEPR polymorphism; however, they observed significantly higher levels of leptin in obese children compared to healthy peers [20]. In analysis performed previously in our department in the population of 46 newly diagnosed ALL patients, we found a strong positive correlation between leptin levels and body mass index, with no difference between leptin values during maintenance therapy and after completion of the treatment. No difference between leptin levels during maintenance therapy and after completion of the treatment was noted [21]. In the present study, leptin concentrations in the control group were statistically higher than in cancer survivors, but predominance of girls in the control group can be responsible for this. This result corresponds with Wasik et al. analysis, which proved significantly higher concentrations of leptin in healthy Polish children, while in leukemic patients this relationship was not present [22]. Our study has not shown any differences in leptin concentrations between boys and girls neither between children in different Tanner stages. It is opposite to Garcia-Mayor et al. analysis in population of healthy children, where they found lower leptin levels in boys than in girls in every pubertal stage [23]. 

Analyzing serum leptin concentrations according to different LEPR genotypes we found statistically higher leptin levels in homozygotic AA alleles carriers, whereas in control group GG homozygotes leptin levels were significantly higher. Multivariate analysis also showed influence of GG genotype on serum leptin levels. Correlations between anthropometric parameters, especially BMI and body mass or fat mass content and Q223R LEPR polymorphism, were the subjects of numerous analyses. Many of them proved that A allele carriage is connected with the higher body mass, tendency to obesity, higher body mass index, and fat tissue content, as well as higher leptin concentration in blood serum, whereas others did not confirm similar correlations [2, 24]. In comprehensive review, Paracchini et al., based on meta-analysis of 18 English-language articles, have not found any correlations between different polymorphisms of the leptin receptor gene (including Q223R, K109R, and K656N) and obesity [1]. Their results explain mainly the polygenic etiology of fat tissue development redundancy. Considine et al. proved that the increasing content of fat tissue comes with the increasing leptin concentration, simultaneously suggesting the contribution of leptin resistance to the pathogenesis of obesity [25]. In population of Polish obese children Pyrzak et al. did not confirme relation between Q223R polymorphism and tendency to obesity [26]. Guízar-Mendoza et al. who analyzed of 103 Mexican adolescents did not find any differences in LEPR Q223R genotypes distribution between obese and nonobese patients [27]. 

The risk of obesity and overweight in ALL survivors is estimated from few percent to even 57%, according to Lughetti et al.'s meta-analysis [10]. In our examined population the frequency of overweight was 16.21% (*n* = 12, 10 boys) and obesity 2.7% (*n* = 2, 2 boys); all of the patients were treated for ALL. While the predominance of A allele was proved to be connected with higher fat mass and body mass index, in our analysis of the group of overweight and obese children we found predominance of allele G (50%), compared to whole analyzed group (28.4%) and controls (39.21%). It can suggest the influence of GG genotype on overweight predisposition. Skoczen et al. in their work performed on 82 Polish ALL survivors found BMI > 24 kg/m^2^ in 31% of patients, more than in our analysis. In their study no correlation between LEPR genotype and leptin levels was reported, although they observed higher concentrations of leptin in obese individuals compared to those with normal weight [28]. Contrary, Ross et al. in their analysis based on 600 ALL survivors found higher frequency of BMI 25 kg/m^2^ in girls homogenous with Arg allele, while similar coincidence was not found in males [11].

While antineoplastic treatment leads to overweight and obesity, cranial radiotherapy (CRT) is proved to be one of the most important factors. Analysis of childhood cancer survivors from Karaman et al. showed significantly higher BMI and leptin levels, especially in girls after CRT, and similar results were achieved by Birkebeak's analysis [29, 30]. However Arguelles et al. showed no differences in serum leptin levels between irradiated and nonirradiated patients, as well as no differences were found between boys and girls [31]. In our analysis we showed higher levels of serum leptin in irradiated compared to nonirradiated subjects. We did not find differences in BMI between these two groups of patients, but in the whole group of cancer survivors we found higher leptin levels in obese subjects compared to nonobese. Taking under consideration the risk of obesity in the stepwise regression model for BMI we found strong connection with fat mass and bone mass, but we did not find any connection between body mass index and leptin concentrations neither with cranial radiotherapy. The small number of patients in the analysis reduces the interpretability of our findings. First Quinton et al. in the group of postmenopausal women, then Ross et al. in his analysis based on 600 individuals confirmed the hypothesis that Arg homozygous female ALL survivors have lower leptin binding affinity [2, 11].

In the present study, we showed no correlations between leptin levels and bone mass in young cancer survivors, but the regression model for leptin demonstrated positive connection between leptin levels and total bone mineral density, but negative connection with bone mass in the lumbar region. Carriage of the GG genotype and cranial radiotherapy amplified this influence. Much research explored relationships between leptin and densitometric parameters such as bone mineral density in the different parts of skeleton and results were highly ambiguous. Most of the analyses were performed in populations of adults. Yamauchi et al. indicated the correlation between BMD and blood concentration of leptin in postmenopausal woman, just like Pasco et al. and oppositely to Klein et al. Responsibility for the differences in the results of the analysis goes to high changeability of leptin secretion and its dependence from stressful, metabolic, or hormonal factors [12–14]. 

Our results did not prove the influence of LEPR genotype on total and spine bone mineral density nor on bone mineral content. Richert's et al. in the group of prepubertal boys showed that the homozygous A allele carriers had higher BMC in comparison with homozygotes without that allele, whereas heterozygotes had middle values. In Richert's study no differences in bone mineral density in different skeleton regions, except femoral diaphysis were shown [9]. Similarly, Crabbe et al. also did not confirm influence of LEPR Q223R genotype on bone mineral mass either [15]. Kim et al. in their analysis on a large group of Korean females before and after menopause did not find any differences in leptin concentration according to Q223R genotype or any correlations between leptin concentration and bone mineral density in various skeletal areas [16]. Oppositely, Koh et al. had proven higher spine BMD among Korean A allele carriers in comparison to those without allele G [17].

To our knowledge, this is the first study exploring the influence of Q223R gene polymorphism on bone mass in young survivors of childhood cancer. This analysis had unfortunately some limitations. Firstly, it was based on small group of individuals, so some of the results can have coincidential character. Secondly, there was a large age range of participants, which may additionally can bring divergent results. Because our findings on relationship between specific genotype and anthropometric parameters correspond with some analyses reported elsewhere also contradict with the results of others; and further prospective research on a larger group of individuals is needed.

## Figures and Tables

**Table 1:** Characteristics of the examined and control group.

	Examined group		Control group		*P *
	*n* = 74		*n* = 51		
	*n *	%	*n *	%	
Gender					
Girls	32	43.24	17	33.33	0.265
Boys	42	56.76	34	66.67	
Diagnosis					
Acute leukemias	64				
Lymphomas	10				
Average age at the beginning of the treatment (years)	6.292 ± 3.685				
Average age at a time of the end of the treatment (years)	8.857 ± 3.537				
Average age at the time of the analysis (years)	15.473 ± 2.643		14.77 ± 3.643		0.881

**Table 2:** Distribution of Q223R polymorphism.

	Examined group		Control group		
Genotype	*n* = 74		*n* = 51		*P *
	*n *	%	*n *	%	
GG	21	28.38	20	39.22	0.205
Girls	6	28.57	9	45.00	0.275
Boys	15	71.43	11	55.00	
Leukemias	19	90.48			
Lymphomas	2	9.52			
GA	42	56.76	24	47.06	0.286
Girls	22	52.38	5	20.83	0.012
Boys	20	47.62	19	79.17	
Leukemias	35	83.33			
Lymphomas	7	16.67			
AA	11	14.86	7	13.72	0.858
Girls	4	36.36	3	42.86	0.783
Boys	7	63.64	4	57.14	
Leukemias	10	90.91			
Lymphomas	1	9.09			

**Table 3:** Values of the anthropometric and densitometric parameters in the whole group, according to Q223R LEPR genotype.

	Whole group	GG	AG	AA	*F *	*P *
WHR^1^	0.8606	0.8489	0.8672	0.8615	0.092	0.912
Height SDS	−0.1974	−0.3383	−0.2210	0.1633	1.407	0.260
BMI SDS^2^	0.556	0.360	0.434	1.357	1.054	0.360
BMD total SDS^3^	−0.0132	0.5292	−0.2691	−0.1386	0.880	0.423
BMD spine SDS^4^	−0.2610	0.0383	−0.4227	−0.2657	0.800	0.457
LEAN SDS^5^	0.1502	0.0180	0.3396	−0.2183	0.281	0.756
FAT SDS^6^	4.7943	6.0488	4.1250	4.7471	0.265	0.769
BMC SDS^7^	0.3244	0.6000	0.1923	0.2671	0.441	0.647
		*P* ^∗1^ = ns	*P* ^∗2^ = ns	*P* ^∗3^ = ns		

^1∗^ *P* value between genotype GG and AG.

^2∗^ *P* value between genotype AA and AG.

^3∗^ *P* value between genotype GG and AA.

^1^WHR: waist to hip ratio.

^2^BMI SDS: Body Mass Index Standard Deviation Score.

^3^BMD total SDS: total Bone Mineral Density Standard Deviation Score.

^4^BMD spine SDS: Bone Mineral Density in the lumbar area Standard Deviation Score.

^5^LEAN SDS: Lean Mass Standard Deviation Score.

^6^FAT SDS: Fat Mass Standard Deviation Score.

^7^BMC SDS: Bone Mineral Content Standard Deviation Score.

**Table 4:** Leptin concentration in the whole examined and control groups, accordingly to Q223R LEPR genotype.

LEPTIN	Examined group		Control group		P*
	Value (ng/mL)	SD ±	Value	SD ±	Sig
Whole group	6.640	7.699	14.484	19.280	0.013
GG	3.78789	3.764	7.528	8.963	0.021
AG	6.63087	8.347	7.608	13.400	0.67
AA	7.83218	7.810	1.696	19.949	0.031

*P value as a comparison in leptin value between examined group and control group.

**Table 5:** Correlation between anthropometric and densitometric parameters in the whole group.

Whole group		Leptin levels	WHR	Height SDS	BMI SDS	BMDt SDS	BMDs SDS	FAT SDS	LEAN SDS	BMC SDS
		*P *								
Leptin levels	Correlation coefficient		0.55	0.263	0.152	0.444	0.748	0.586	0.409	0.686
WHR		−0.079		0.64	0.024	0.927	0.259	0.613	0.309	0.309
Height SDS		−0.207	−0.08		0.406	0.033	0.003	0.286	0	0
BMI SDS		0.264	**0.371**(∗)	0.133		0.003	0.001	0	0.041	0
BMDt SDS		0.143	−0.414	**0.334**(∗)	**0.459**(∗)		0.023	0.006	0.006	0.023
BMDs SDS		−0.06	0.142	**0.447**(∗)	**0.500**(∗)	**0.749**(∗)		0.005	0	0
FAT SDS		0.102	0.272	0.171	**0.651**(∗)	**0.424**(∗)	**0.429**(∗)		0.282	0
LEAN SDS		−0.154	−0.262	**0.742**(∗)	**0.321**(∗)	**0.424**(∗)	**0.562**(∗)	0.172		0
BMC SDS		0.076	−0.262	**0.624**(∗)	**0.651**(∗)	**0.803**(∗)	**0.782**(∗)	**0.561**(∗)	**0.703**(∗)	

**Table 6:** Correlation between anthropometric and densitometric parameters in the group with LEPR gene genotype GA.

GA		Leptin levels	WHR	Height SDS	BMI SDS	BMDt SDS	BMDs SDS	FAT SDS	LEAN SDS	BMC SDS
		*P *								
Leptin levels	Correlation coefficient		0.696	0.321	0.094	0.963	0.309	0.305	0.66	0.926
WHR		−0.08		0.977	0.483	0.781	0.474	0.323	0.582	0.782
Height SDS		−0.256	0.007		0.871	0.032	0.003	0.799	0	0
BMI SDS		0.42	0.166	0.037		0.004	0.032	0	0.369	0.011
BMDt SDS		0.012	0.066	**0.459**(∗)	**0.582**(∗)		0	0.072	0.003	0
BMDs SDS		−0.262	−0.17	**0.608**(∗)	**0.457**(∗)	**0.738**(∗)		0.223	0	0
FAT SDS		0.265	0.233	0.058	**0.842**(∗)	0.391	0.27		0.974	0.109
LEAN SDS		−0.115	−0.131	**0.817**(∗)	0.201	**0.596**(∗)	**0.727**(∗)	0.007		0
BMC SDS		−0.025	−0.066	**0.703**(∗)	**0.530**(∗)	**0.891**(∗)	**0.844**(∗)	0.351	**0.837**(∗)	

**Table 7:** Correlation between anthropometric and densitometric parameters in the group with LEPR gene genotype AA.

AA		Leptin levels	WHR	Height SDS	BMI SDS	BMDt SDS	BMDs SDS	FAT SDS	LEAN SDS	BMC SDS
		*P *								
Leptin levels	Correlation coefficient		0.76	0.188	1	0.873	1	1	0.873	0.624
WHR		0.143		0.957	0.125	0.208	0.544	0.872	0.872	0.156
Height SDS		0.7	0.029		0.09	0.094	0.148	0.094	0.036	0.036
BMI SDS		0	0.696	0.685		0.006	0.006	0.09	0.078	0.001
BMDt SDS		0.1	0.6	0.679	**0.901**(∗)		0.036	0.119	0.253	0
BMDs SDS		0	0.314	0.607	**0.901**(∗)	**0.786**(∗)		0.007	0.119	0.023
FAT SDS		0	0.086	0.679	0.685	0.643	**0.893**(∗)		0.148	0.094
LEAN SDS		−0.1	0.086	**0.786**(∗)	0.703	0.5	0.643	0.607		0.119
BMC SDS		0.3	0.657	**0.786**(∗)	**0.955**(∗)	**0.964**(∗)	**0.821**(∗)	0.679	0.643	

**Table 8:** Correlation between anthropometric and densitometric parameters in the group with LEPR gene genotype GG.

GG		Leptin levels	WHR	Height SDS	BMI SDS	BMDt SDS	BMDs SDS	FAT SDS	LEAN SDS	BMC SDS
		*P *								
Leptin levels	Correlation coefficient		0.762	0.242	0.932	0.308	0.937	0.356	0.332	0.831
WHR		−0.082		0.526	0.002	0.612	0.937	0.06	0.631	0.026
Height SDS		−0.435	−0.215		0.632	0.983	0.712	0.329	0.007	0.221
BMI SDS		−0.03	**0.827**(∗)	0.154		0.983	0.762	0	0.175	0.001
BMDt SDS		0.383	0.173	−0.007	0.007		0.101	0.681	0.681	0.216
BMDs SDS		0.027	0.582	−0.119	0.098	0.497		0.762	0.931	0.47
FAT SDS		−0.35	0.582	0.309	**0.888**(∗)	0.133	0.098		0.124	0
LEAN SDS		−0.367	0.164	**0.726**(∗)	0.42	0.133	−0.028	0.469		0.103
BMC SDS		−0.08	**0.664**(∗)	0.381	**0.809**(∗)	0.497	0.231	**0.851**(∗)		

**Table 9:** Stepwise forward regression model for BMI value.

Parameter	*b *	SD	*t *	*P *
BMD total SDS	0.2812	0.0955	2.9942	**0.0146**
BMD spine SDS	0.1854	0.1641	1.1299	0.2488
FAT SDS	0.1942	0.0345	5.6162	**0.0002**

**Table 10:** Stepwise forward regression model for leptin levels.

Parameter	*b *	SD	*t *	*P *
BMD total SDS	5216.33	893.233	5.8398	**0.00024**
BMD spine SDS	−6526.86	1303.73	−5.006	**0.00073**
LEAN SDS	−2477.40	986.33	−2.5117	**0.0033**
GG genotype	3755.55	2014.82	1.8639	0.0952
Cranial radiotherapy	2191.25	2031.74	1.0785	0.3088
